# Self-reported long COVID associated with sleep problems and poor sleep quality among adults in Germany: results of the German Health Update 2023 (GEDA 2023)

**DOI:** 10.3389/fpubh.2026.1814427

**Published:** 2026-10-14

**Authors:** Ann-Kristin Beyer, Dinara Yessimova, Lisa Kümpel, Grit Eckert, Robert Schlack, Hannelore Neuhauser, Giselle Sarganas, Julia Nübel

**Affiliations:** Department of Epidemiology and Health Monitoring, Robert Koch Institute, Berlin, Germany

**Keywords:** long COVID, post COVID-19 condition, post-acute sequelae of COVID-19, problems maintaining sleep, sleep onset problems, sleep problems, sleep quality

## Abstract

**Introduction:**

Long COVID refers to various long-term health impairments following a SARS-CoV-2 infection, including physical, cognitive, and psychological symptoms. Sleep problems are among the most prevalent neuropsychiatric symptoms of long COVID and can negatively affect daily functioning and quality of life. This study aims to investigate sleep onset problems, problems maintaining sleep and poor sleep quality in associations with long COVID among adults in Germany.

**Methods:**

Analyses are based on data from the cross-sectional nationwide telephone interview survey “German Health Update” (GEDA 2023). Between March 2023 and February 2024, 9,310 adults (50.8% female, median age = 60.8 years) answered questions on any prior SARS-CoV-2 infection and long COVID status, as well as on sleep onset problems, problems maintaining sleep and poor sleep quality over the past 4 weeks. Weighted prevalence estimates for each sleep variable were calculated by self-reported SARS-CoV-2 infection and long COVID status. Multivariable logistic regressions were conducted, adjusted for age, sex, level of education, chronic conditions, depressive symptoms, COVID-19 vaccination status, and multiple SARS-CoV-2 infections.

**Results:**

Participants with self-reported current long COVID reported higher proportions of sleep onset problems (32.6%), problems maintaining sleep (47.8%) and poor sleep quality (44.8%) compared to participants without a SARS-CoV-2 infection (20.0%, 30.8 and 24.1%, respectively) or prior infected participants without long COVID (13.8%, 23.8 and 20.0%, respectively). In multivariable logistic regression analyses, participants with self-reported current long COVID had an increased likelihood of experiencing problems maintaining sleep (adjusted odds ratio (aOR) = 1.73; 95% CI = 1.23–2.44) and a higher likelihood of poor sleep quality (aOR = 1.81; 95% CI = 1.29–2.55) compared to non-infected individuals; however, sleep onset problems were not associated with long COVID.

**Discussion:**

Our findings from general population data suggest that adults with current long COVID have 1.7- to 1.8-fold higher odds of problems maintaining sleep and poor sleep quality than non-infected individuals. Given the potential impact on quality of life and physical and psychological functioning, sleep-related symptoms may represent an important target for clinical assessment and management in individuals with long COVID.

## 1 Introduction

The Coronavirus Disease 2019 (COVID-19) pandemic had far-reaching global consequences with long-lasting impact on people’s health. While most people typically recover quickly after SARS-CoV-2 infection, some may experience health consequences more than 4 weeks after infection, referred to as post-acute sequelae of COVID-19 (PASC), commonly known as long COVID (1). Long COVID can manifest as a multi-organ disease, presenting a wide range of health complaints and symptoms that vary in their clinical characteristics, severity and progression (2–4). Long COVID comprises the “post COVID-19 condition”, which is defined as continuation or development of new symptoms 3 months after the initial SARS-CoV-2 infection, with these symptoms lasting for at least 2 months with no other explanation by World Health Organization (WHO) (5) and has become an increasingly important public health concern (6) with negative impact on daily functioning and quality of life (7).

People with long COVID experience persistent or newly developed physical, cognitive or psychological symptoms, such as fatigue, respiratory symptoms (i.e., cough and shortness of breath) or cognitive impairment (5, 8–11). Sleep problems are among the most prevalent neuropsychiatric symptoms of long COVID (12–14). However, the frequency of reported sleep problems varies considerably. A systematic review and meta-analysis of 153 studies involving 252,437 COVID-19 survivors showed that the pooled prevalence of sleep problems that persist beyond 28 days after acute SARS-CoV-2 infection was 29% (15), while other systematic reviews and meta-analyses reported even higher prevalence of sleep problems of approximately 45% (12, 16). This variability in prevalence depends on study characteristics, different definitions or type of sleep problems. For instance, studies may investigate specific subgroups that are not representative of the general population due to selection bias (e.g., people hospitalized due to COVID-19) or general sleep problems that do not sufficiently address differentiated associations between various sleep problems and long COVID. Furthermore, studies frequently did not include a control group of uninfected subjects (8, 10). However, a comparison of those infected with SARS-CoV-2 with non-infected controls is needed in order to distinguish SARS-CoV-2-induced sleep problems from the effects of comorbid health problems, since sleep problems are not specific for long COVID but highly prevalent in the general population (17)—and several physical or psychiatric disorders can also have a negative impact on sleep. For example, Xu et al. (8) found in a meta-analysis that the pooled prevalence of sleep problems in SARS-CoV-2-infected individuals 3 months after infection was 12.2%, only 3.5 percentage points higher than in the control group without infection (8.7%). Additionally, potential confounding of sociodemographic and other health-related factors (including pre-existing conditions) or SARS-CoV-2 associated information has not been fully explored in previous studies. However, particularly higher age, female sex, lower education, or conditions such as depression were shown to be associated with a higher risk of sleep problems (18–21). Factors such as pre-existing chronic conditions, COVID-19 vaccination, or multiple SARS-CoV-2 infections may also influence the risk of long COVID associated symptoms and sleep outcomes (15).

Therefore, the aim of this study is to examine the association between SARS-CoV-2 infection and long COVID with sleep problems among adults in Germany. The study focuses on the frequently reported problems of difficulty falling asleep and difficulty staying asleep, as well as poor sleep quality in order to investigate the links between sleep and long COVID in greater detail. Furthermore, the present analyses are based on nationwide population-based data from Germany and consider control groups and further influencing factors.

## 2 Methods

### 2.1 Study design and sample

The nationwide cross-sectional survey German Health Update 2023 (GEDA 2023) collected information on the health situation of the resident population aged 15 and over living in Germany between March 2023 and February 2024. The data of this observational study were collected using a standardized computer-assisted telephone interview (22). Participants were selected using the dual-frame method with a random sample of landline and mobile phone numbers (23). The monthly response rate was calculated according to the standards of the American Association for Public Opinion Research [AAPOR (24)] and varied between 17.4 and 19.2%.

The present analyses are based on 9,926 GEDA 2023 participants aged 18 years and older who were asked questions about their health status and influencing factors. After excluding participants with missing information on sleep problems, SARS-CoV-2 infection or long COVID status, as well as sociodemographic, health-related or SARS-CoV-2 associated variables (*n* = 391), and whose last SARS-CoV-2 infection was less than 4 weeks ago (*n* = 225), the final study sample comprised 9,310 participants.

### 2.2 Variables

#### 2.2.1 Sleep problems and sleep quality

Sleep onset problems and problems maintaining sleep were assessed by asking participants how often they had problems falling asleep and staying asleep in the last 4 weeks. The response options for the two questions were “not at all during the past four weeks”, “less than once a week”, “once or twice a week”, and “three times a week or more”. Answers were dichotomized for both questions, with an occurrence of three times or more a week indicating sleep onset problems or problems maintaining sleep according to the time criterion of the International Statistical Classification of Diseases and Related Health Problems [ICD-10 (25), F51.0]. Sleep quality was assessed using an adapted question from the Pittsburgh Sleep Quality Index [PSQI (26); German version: Riemann and Backhaus (27)] “How would you rate the quality of your sleep during the past four weeks?” with the response options “very good,” “quite good,” “quite poor” or “very poor.” The options “quite poor” and “very poor” were categorized into “poor sleep quality” and the response options “very good” and “quite good” into “good sleep quality”. The distribution of ordinal sleep variables was also estimated and presented in the Supplementary Table S1.

#### 2.2.2 Self-reported SARS-CoV-2 infection and long COVID status

Participants were asked whether they had or ever had one or more SARS-CoV-2 infections or COVID-19 diseases. If yes, participants were further asked if they think they have or ever had long COVID or a post COVID-19 condition, and whether they have been diagnosed by a doctor with long COVID or a post COVID-19 condition. If they confirmed either subjective or medically diagnosed long COVID/post COVID-19 condition, they were further asked whether long COVID or the post COVID-19 condition currently still exists. According to their self-reported SARS-CoV-2 infection and long COVID status, participants were categorized into four groups: “no SARS-CoV-2 infection”,” prior SARS-CoV-2 infection without long COVID”, “prior SARS-CoV-2 infection and previous long COVID”, and “prior SARS-CoV-2 infection and current long COVID”.

#### 2.2.3 Covariates

For sociodemographic information, sex assigned at birth (female or male), age in years (grouped into 18–29 years, 30–44 years, 45–64 years, 65–79 years, 80 years and over), and level of education according to Comparative Analysis of Social Mobility in Industrial Nations (CASMIN) classification with low, medium or high (28) were assessed.

Health-related information included chronic conditions and depressive symptoms. Chronic conditions were measured using the question: “Do you have a chronic condition or a long-term health problem? This refers to health conditions or problems which have lasted, or are expected to last, for six months or more”, with response options “yes” and “no.” Depressive symptoms were assessed with the Patient Health Questionnaire-2 [PHQ-2 (29)], assessing the main symptoms of major depression according to the Diagnostic and Statistical Manual of Mental Disorders [DSM-IV, 4th edition (30)]. Participants were asked “Over the last two weeks, how often have you been bothered by any of the following problems? (a) Little interest or pleasure in doing things, and (b) Feeling down, depressed, or hopeless?”. Response options were “not at all” (0), “several days” (1), “more than half the days” (2) and “nearly every day” (3). A total score of three points or more was defined as “depressive symptoms.”

For SARS-CoV-2 associated information, COVID-19 vaccination status was assessed using the question “Have you already been vaccinated against the coronavirus disease COVID-19?” with response options “yes” and “no.” To measure multiple SARS-CoV-2 infections, participants with self-reported SARS-CoV-2 infection were asked “How many SARS-CoV-2 infections or COVID-19 diseases have you had?”. Responses were recoded into “yes” for multiple (two or more) SARS-CoV-2 infections and “no” for less than two infections.

### 2.3 Statistical analyses

Weighted prevalence estimates for each sleep variable were calculated depending on SARS-CoV-2 infection and long COVID status. 95% confidence intervals (CIs) were derived using design-based standard errors (Wald-type intervals). To investigate the association between sleep problems and long COVID, effect estimates from logistic regression models were calculated: an unadjusted model and a model adjusted for sociodemographic indicators (age group, sex, and level of education), health-related indicators (chronic conditions and depressive symptoms), and SARS-CoV-2 associated information (COVID -19 vaccination status and multiple SARS-CoV-2 infections). Thus, the adjusted model accounts for key factors that are commonly considered as potential confounders of the association between long COVID status and sleep outcomes. These variables were selected to reduce potential confounding, as identified in previous studies (15, 18–21). Non-infected individuals were used as reference group throughout all analyses. Models were assessed and selected for relative comparison using the survey log-likelihood and a corresponding Bayesian Information Criterion (BIC)-like information criterion. Across all three sleep outcomes, adding sociodemographic, health-related and SARS-CoV-2 associated covariates consistently improved model fit relative to the unadjusted model. In addition, the goodness-of-fit of the fully adjusted model was assessed using a design-based Hosmer-Lemeshow-type test appropriate for survey-weighted logistic regression models (31). In order to examine whether the observed associations between long COVID and sleep problems differed depending on sex, we tested for two-way interaction effects for SARS-CoV-2 infection and long COVID status with sex in the unadjusted model for each sleep variable. As no significant interactions were found in the unadjusted models (interaction effects for sleep onset problems: *p* = 0.562; for problems maintaining sleep: *p* = 0.084; for poor sleep quality: *p* = 0.109), the respective interaction terms were not included in the final regression models.

Statistical significance was evaluated based on non-overlapping confidence intervals derived using design-based standard errors (Wald-type intervals) or *p*-values derived from logistic regression models, using a conventional significance level of 5% (32). A survey-specific weighting factor was applied to all analyses to account for the probability of the participants being drawn and to adjust the survey sample to the sociodemographic-geographic population structure of Germany according to official statistics with regard to sex, age, federal state (Federal Statistical Office as at 31.12.2020) and education [Microcensus 2018, according to International Standard Classification of Education, ISCED; (23, 33)]. All analyses were conducted with R version 4.3.0 (34) using RStudio version 2024.4.0.735 (35). Survey-weighted generalized linear models (*svyglm*, binomial family) from the *survey* package (version 4.4.2), applied to a survey design object incorporating the GEDA 2023 weights, were used. The design-based standard errors produced by *svyglm* are analogous to robust/sandwich estimators in the non-survey setting.

### 2.4 Sensitivity analysis

To assess the robustness of the results, a more restrictive variable for SARS-CoV-2 infection and long COVID status was applied for reclassification as part of a sensitivity analysis. Therefore, the four-level variable for SARS-CoV-2 infection and long COVID status was based solely on medically diagnosed long COVID/post COVID-19 condition, without considering subjective long COVID/post COVID-19 condition. In addition, participants whose last SARS-CoV-2 infection occurred less than twelve weeks prior were excluded to align with the WHO’s clinical case diagnostic criteria. This more restrictive approach aimed to compare the results of the main analyses with those based on a more rigorous long COVID definition.

## 3 Results

The analysis sample comprised 9,310 adults (median age = 60.8 years); 50.8% were female. The majority had a medium level of education (53.7%, low, 26.7%, high: 19.7%). 51.0% reported having a chronic condition and 17.1% depressive symptoms. 90.8% received a COVID-19 vaccination and 17.5% reported multiple SARS-CoV-2 infections. About a third of participants (36.5%) reported that they never had a SARS-CoV-2 infection. Every second participant (50.0%) reported a prior SARS-CoV-2 infection without long COVID, 6.9% reported a prior SARS-CoV-2 infection and previous long COVID and 6.5% reported current long COVID (see Table 1). Sample characteristics stratified by SARS-CoV-2 infection and long COVID status are depicted in the Supplementary Table S2.

**Table 1:** Sample characteristics.

Variable	Total (*N* = 9,310)		
	*n*	%	(95% CI)
Age groups			
18–29 years	792	16.3	(14.9–17.8)
30–44 years	1,374	22.4	(20.9–24.0)
45–64 years	3,413	34.5	(33.0–36.0)
65–79 years	2,557	18.2	(17.1–19.2)
80 years and older	1,174	8.5	(7.8–9.3)
Sex			
Female	4,944	50.8	(49.2–52.5)
Male	4,366	49.2	(47.5–50.8)
Level of education (CASMIN)			
Low	1,429	26.7	(25.1–28.3)
Medium	4,035	53.7	(52.0–55.3)
High	3,846	19.7	(18.7–20.6)
Chronic condition			
Yes	4,933	51.0	(49.3–52.6)
No	4,377	49.0	(47.4–50.7)
Depressive symptoms			
Yes	1,260	17.1	(15.7–18.4)
No	8,050	82.9	(81.6–84.3)
COVID-19 vaccination status			
Yes	8,693	90.8	(89.7–91.9)
No	617	9.2	(8.1–10.3)
Multiple SARS-CoV-2 infections			
Yes	1,442	17.5	(16.2–18.8)
No	7,868	82.5	(81.2–83.8)
SARS-CoV-2 infection and long COVID status			
No infection	3,470	36.5	(34.9–38.1)
Infection without long COVID	4,732	50.0	(48.4–51.7)
Previous long COVID	566	6.9	(6.0–7.8)
Current long COVID	542	6.5	(5.6–7.4)

*n* unweighted, % weighted; CI, Confidence interval; CASMIN, Comparative Analysis of Social Mobility in Industrial Nations.

### 3.1 Frequency of sleep problems

Prevalence estimates of self-reported sleep problems and poor sleep quality varying by SARS-CoV-2 infection and long COVID status are depicted in Table 2.

**Table 2:** Weighted prevalence estimates of sleep onset problems, problems maintaining sleep and poor sleep quality overall and varying by SARS-CoV-2 infection and long COVID status, as well as effect estimates from logistic regression analyses.

Variable	Prevalence		Effect estimates			
	*n*	*%* (95% CI)	OR (95% CI)	*p*	aOR (95% CI)	*p*
Sleep onset problems	1,358	17.8 (16.4–19.1)				
No infection	587	20.0 (17.8–22.3)	Ref.		Ref.	
Infection without long COVID	530	13.8 (12.2–15.5)	0.64 (0.52–0.78)	<0.001	0.79 (0.62–0.99)	0.043
Previous long COVID	94	20.9 (15.7–27.3)	1.06 (0.72–1.54)	0.772	1.09 (0.74–1.60)	0.670
Current long COVID	147	32.6 (25.9–40.2)	1.94 (1.36–2.77)	<0.001	1.38 (0.92–2.05)	0.118
Problems maintaining sleep	2,727	28.4 (26.9–29.9)				
No infection	1,094	30.8 (28.4–33.4)	Ref.		Ref.	
Infection without long COVID	1,204	23.8 (21.9–25.8)	0.70 (0.60–0.82)	<0.001	0.92 (0.77–1.09)	0.333
Previous long COVID	175	30.6 (24.8–37.0)	0.99 (0.72–1.35)	0.935	1.11 (0.77–1.58)	0.575
Current long COVID	254	47.8 (40.8–54.9)	2.05 (1.51–2.79)	<0.001	1.73 (1.23–2.44)	0.002
Poor sleep quality	1,934	23.6 (22.1–25.0)				
No infection	729	24.1 (21.8–26.6)	Ref.		Ref.	
Infection without long COVID	860	20.0 (18.2–22.0)	0.79 (0.66–0.94)	0.008	0.87 (0.71–1.07)	0.182
Previous long COVID	125	26.0 (20.4–32.4)	1.10 (0.79–1.55)	0.566	1.06 (0.73–1.53)	0.751
Current long COVID	220	44.8 (37.9–52.0)	2.55 (1.86–3.50)	<0.001	1.81 (1.29–2.55)	<0.001

OR, odds ratio; aOR, adjusted odds ratio; adjusted for age group, sex, level of education, chronic conditions depressive symptoms, COVID-19 vaccination status and multiple SARS-CoV-2 infections; CI, Confidence interval.

The overall prevalence of sleep onset problems, problems maintaining sleep, and poor sleep quality during the last 4 weeks was 17.8% (95% CI = 16.4–19.1), 28.4% (95% CI = 26.9–29.9), and 23.6% (95% CI = 22.1–25.0), respectively. The group with a history of SARS-CoV-2 infection and current long COVID consistently displayed the highest prevalence for the three self-reported aspects of sleep problems. Participants with current long COVID reported a higher proportion of sleep onset problems (32.6%; 95% CI = 25.9–40.2), compared to participants who reported no SARS-CoV-2 infection or prior SARS-CoV-2 infection without long COVID. In addition, participants with current long COVID were more frequently affected by self-reported problems maintaining sleep (47.8%; 95% CI = 40.8–54.9) and by poor sleep quality (44.8%; 95% CI = 37.9–52.0) than the other three groups (Figure 1).

**Figure 1:**
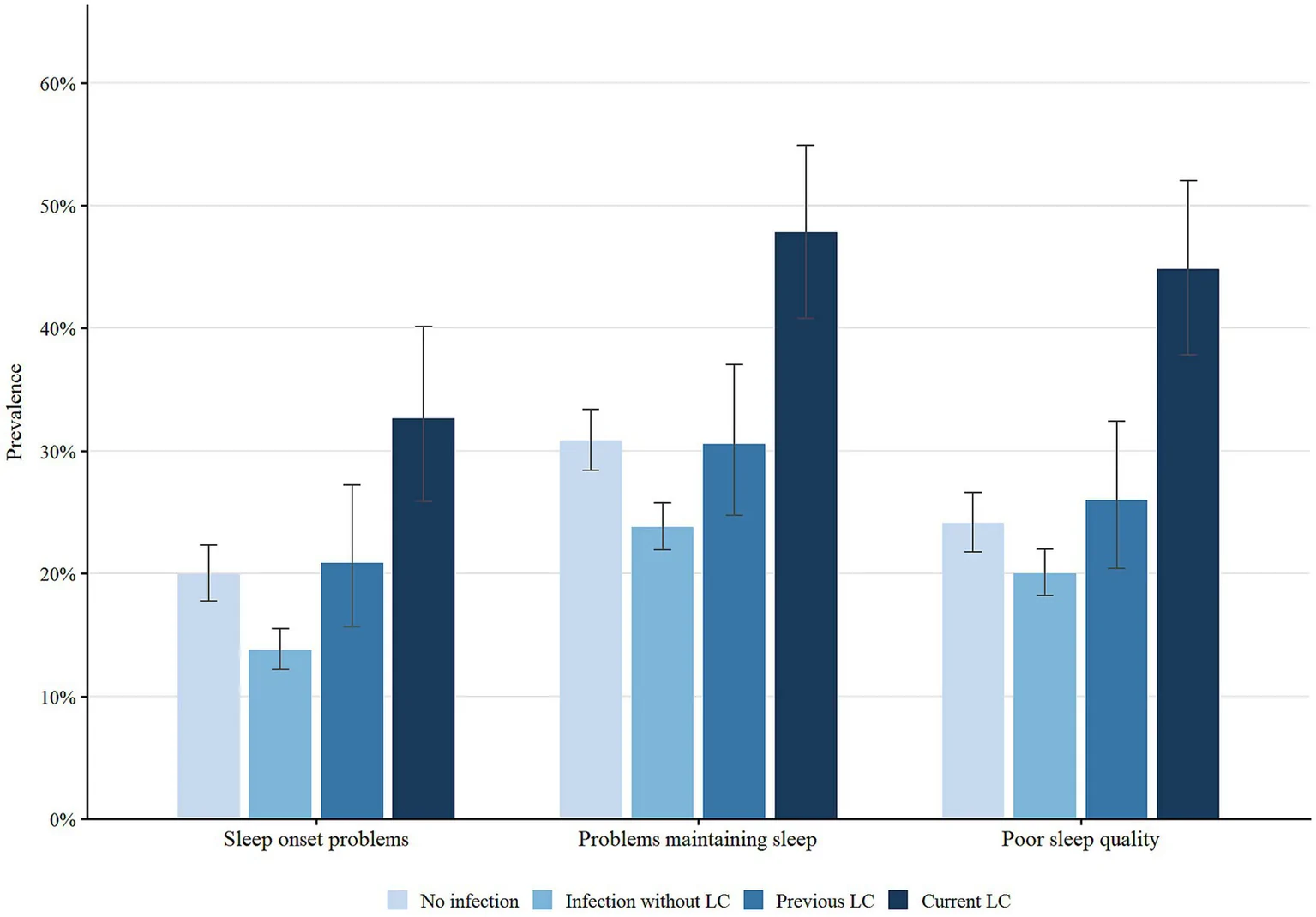
Weighted prevalence estimates and 95% confidence intervals of sleep onset problems, problems maintaining sleep, and poor sleep quality, varying by SARS-CoV-2 infection and long COVID status. LC, Long COVID.

The prevalence of participants reporting all three sleep problems simultaneously according to SARS-CoV-2 infection and long COVID status is provided in the Supplementary Table S3.

### 3.2 Multivariable logistic regression analyses

Effect estimates from logistic regression analyses are presented in Table 2. In the unadjusted models, current long COVID was significantly associated with a higher likelihood for self-reported sleep onset problems (odds ratio (OR) = 1.94; 95% CI = 1.36–2.77), problems maintaining sleep (OR = 2.05; 95% CI = 1.51–2.79) and poor sleep quality (OR = 2.55; 95% CI = 1.86–3.50), compared to non-infected participants. After adjusting for sociodemographic information, health-related and SARS-CoV-2 associated variables, associations of self-reported sleep problems with current long COVID were weakened but remained significant for problems maintaining sleep (adjusted odds ratio (aOR) = 1.73; 95% CI = 1.23–2.44) and poor sleep quality (aOR = 1.81; 95% CI = 1.29–2.55). Thus, the odds of problems maintaining sleep and poor sleep quality were around 70–80% higher among participants with current long COVID compared to non-infected individuals. The full model outputs including all covariates are displayed in Supplementary Table S4. Goodness-of-fit tests indicated no evidence of lack of fit for any of the three outcomes (all *p* > 0.05).

In addition, a prior SARS-CoV-2 infection without long COVID significantly reduced the likelihood for each self-reported sleep problem in the unadjusted models, with OR ranging from 0.64 to 0.79. However, in the adjusted models, there was only a significant association between prior SARS-CoV-2 infection without long COVID and sleep onset problems. Furthermore, there were no significant associations between previous long COVID and sleep problems in any of the models, compared to non-infected individuals (Table 2).

### 3.3 Sensitivity analysis

When only self-reported medically diagnosed long COVID status was considered as more restricted long COVID definition, a lower frequency of prior SARS-CoV-2 infection and previous long COVID (*n* = 168; 2.2%) and current long COVID (*n* = 107; 1.3%) was found, while more individuals were categorized into the group with SARS-CoV-2 infection and without long COVID (*n* = 5,184; 58.6%). However, the sensitivity analysis confirmed the robustness of the main findings, showing consistent directions and strengths of the associations in the logistic regression models (see Supplementary Table S5). In the adjusted model, current long COVID was significantly associated with a higher OR for self-reported problems maintaining sleep (aOR = 2.26; 95% CI = 1.11–4.61) and poor sleep quality (aOR = 2.25; 95% CI = 1.07–4.76) compared to non-infected individuals. No other associations were significant, including the association between current long COVID and sleep onset problems.

## 4 Discussion

The aim of the present study was to examine associations between long COVID and sleep onset problems, problems maintaining sleep or poor sleep quality in the general adult population in Germany. Based on four groups, comparisons of the prevalence of sleep problems were possible between those who currently experienced long COVID compared to those who either recovered from long COVID, never experienced long COVID after a SARS-CoV-2 infection or were never infected with SARS-CoV-2.

Our results are consistent with recent findings showing that a significant proportion of individuals with long COVID experience sleep problems (12, 15, 16). Among adults in Germany with current long COVID, we found that the proportions varied depending on the specific sleep problem: one in three reported sleep onset problems and almost half reported problems maintaining sleep and poor sleep quality. Furthermore, our findings are in line with meta-analyses of controlled studies, indicating a higher risk for long COVID associated sleep problems compared to non-infected controls (8, 10). However, our analysis comprises several direct comparison groups with more nuanced results: Problems maintaining sleep and poor sleep quality were more common among participants with current long COVID than in all other comparison groups, while sleep onset problems were more common among participants with current long COVID than in participants without SARS-CoV-2 infection or without long COVID. Notably, findings from the RECOVER study further support the relevance of differentiating between specific sleep-related symptoms in the context of long COVID. In the original analysis including non-infected controls, general “sleep disturbance” was not among the symptoms contributing to the PASC score but was among the non-score symptoms most highly correlated with score-contributing symptoms, suggesting that it may nevertheless represent a relevant long COVID associated outcome (36). More recently, an updated RECOVER analysis identified “snoring or sleep apnea” as a sleep-related symptom contributing to the refined PASC research index (37).

In a general population sample, our results showed that sleep problems and poor sleep quality also occur in individuals who have not been infected with SARS-CoV-2, have never experienced long COVID or those with previous long COVID − albeit less frequent. These findings emphasize that sleep problems are the result of a complex process influenced by a range of determinants and are not specific to long COVID. These include demographic, behavioral, work- and living environment-related or health-related determinants, but also indirect effects of the pandemic (38, 39). However, adjusted multivariable logistic regression analyses revealed that problems maintaining sleep and a poor sleep quality are clearly associated with long COVID, independent of factors such as age, sex, level of education, chronic conditions, depressive symptoms, COVID-19 vaccination status or multiple SARS-CoV-2 infections. Regarding sleep onset problems, however, the association with current long COVID was no longer significant in the adjusted model. Further analyses revealed that a significant association existed in the multivariable regression model when depressive symptoms were not included as covariate (aOR of current long COVID in the adjusted model for sleep onset problems without depressive symptoms = 1.48; 95% CI = 1.02–2.14; *p* = 0.039). Depression, however, often involves sleep onset problems, which is consistent with findings that sleep disorders frequently co-occur with depression (21).

Furthermore, individuals with previous long COVID who have recovered and are no longer affected by long COVID did not show an association with sleep problems or poor sleep quality in all statistical models. The frequency of sleep problems and poor sleep quality among these participants was comparable to that of participants without prior SARS-CoV-2 infection or long COVID, suggesting that full recovery from sleep problems is possible for long COVID patients. However, the group of participants who recovered from long COVID had a smaller sample size than the groups of non-infected participants and those infected without long COVID (*n* = 566 vs. *n* = 3,470 and *n* = 4,732), which lead to wider confidence intervals and greater uncertainty in the corresponding prevalence estimates. Therefore, this finding should be interpreted with caution. In addition, because the sleep outcomes examined were relatively common in the study population, the odds ratios reported here are larger than the corresponding relative risks and should therefore be interpreted as measures of association rather than as direct estimates of relative risk.

In addition, SARS-CoV-2 infection without long COVID unexpectedly had a protective effect on sleep problems. However, this specific group may have been constituted by generally healthier individuals with less comorbidity (see Supplementary Table S2) and milder COVID-19 courses. Accordingly, associations clearly diminished during adjustment and, in the fully adjusted model, only remained significant with regard to sleep onset problems. Since pre-existing conditions as well as other potential confounders (including health behaviors and severity of the acute COVID-19) could not be sufficiently addressed in our study, the observed counterintuitive effect may be attributable to residual confounding.

### 4.1 Strengths, limitations and future directions

A major strength of the present analyses is the large and nationwide population-based sample of German adults, which allows for the detailed analysis and comparison of different unselected groups of community-dwelling adults with and without a history of SARS-CoV-2 infection and long COVID. In addition, the study focuses in particular on sleep onset problems, problems maintaining sleep, and poor sleep quality as major sleep issues associated with long COVID. It also considers further potential confounding factors to investigate associations independently of sociodemographic variables or the effects of comorbid health problems and SARS-CoV-2 associated information such as vaccinations or reinfections. Overall, our study extends previous findings and provides a more nuanced understanding of the associations between sleep and long COVID.

However, there are several limitations that should be noted. The direction of associations and temporal relationships between long COVID and sleep problems could not be evaluated due to the cross-sectional study design of GEDA 2023. Although a recent study showed that long COVID is prospectively associated with sleep problems and poor sleep quality over 3 years (40), there is also evidence of a bidirectional relationship between sleep problems and long COVID (41, 42). A meta-analysis found that pre-existing sleep problems also increased the risk of long COVID (43). This may not have been adequately addressed in our study since pre-existing morbidity was only generally considered in terms of self-reported chronic conditions in the 6 months prior to the interview. Therefore, further longitudinal studies or studies that focus on causal relationships are needed to determine strength and directionality of the association between long COVID and sleep problems. Accounting for pre-existing sleep problems would also allow us to differentiate between sleep problems associated with long COVID and those that existed beforehand. Furthermore, several factors may contribute to sleep problems, especially in relation to SARS-CoV-2 infection or long COVID (15, 44, 45). In our analyses, confounders such as sociodemographic, health-related and SARS-CoV-2 associated factors were taken into account. However, unmeasured confounders (e.g., lifestyle factors, stress, a pre-existing diagnosis of a sleep disorder, or the use of sleep medication), further SARS-CoV-2 infection associated information (e.g., COVID-19 severity, hospitalization or time since SARS-CoV-2 infection) or other specific comorbidities including sleep-related chronic conditions (e.g., cardiovascular diseases) may also have an influence on the associations between long COVID and sleep problems. This should be considered in future studies. In addition, the exact mechanisms underlying the relationship between long COVID and sleep problems remain unclear. These mechanisms are likely multifactorial and may involve a complex interplay of physiological, psychological, and behavioral factors. Persistent inflammation, dysregulation of the immune system, and neurocognitive dysfunction associated with long COVID may also play a role (46, 47). A better understanding of the relevant pathomechanisms may contribute to identifying the most discriminative symptoms as well as the development of targeted and effective care strategies for addressing sleep problems among individuals with long COVID. This should be explored in further research.

The data are based on self-report measures, which may be prone to subjectivity and recall bias and were not assessed by standardized questionnaires and diagnostic criteria. The GEDA study assessed sleep problems using three single items, limiting reliability and failing to capture the full spectrum of sleep disturbances. In particular, other relevant sleep-related symptoms and consequences, such as unrefreshing sleep—which is relevant in the context of myalgic encephalomyelitis / chronic fatigue syndrome (ME/CFS)—and daytime sleepiness were not assessed. Future studies should consider a broader spectrum of sleep-related symptoms and their daytime consequences to provide a more comprehensive understanding of sleep problems associated with long COVID. Although responses were dichotomized according to the ICD-10 time criterion (F51.0), this does not constitute a formal ICD-10 diagnosis. Furthermore, self-reported long COVID was only based on general subjective impression, without information as to whether the complaints occurred at least 4 weeks after infection and thus, actually meet the time criterion of the long COVID definition according to the guidelines by the British National Institute for Health and Care Excellence (NICE) (1). This may lead to a possible overrepresentation of the long COVID cohort, compared to results from studies using more rigorous long COVID definitions, such as the WHO clinical case definition or a medical diagnosis. However, a sensitivity analysis with a more restrictive variable for SARS-CoV-2 infection and long COVID status based on medically diagnosed long COVID supported our findings. Moreover, the stricter, medically diagnosed long COVID definition used in the sensitivity analysis yielded consistently higher effect estimates for current long COVID across all three sleep outcomes. This pattern suggests that our broader and more permissive long COVID definition, applied in the main analysis, may dilute the true association with sleep outcomes. Future research should, therefore, incorporate standardized questionnaires or objective measures of SARS-CoV-2 infection, long COVID, and sleep problems, such as medical records or wearable sleep trackers, which might strengthen the reliability of the data. In addition, at the time of the survey, one-third of the participants reported not having had a SARS-CoV-2 infection yet. However, it is conceivable that there were unnoticed infections, especially in asymptomatic COVID-19 cases, which could have led to misclassification in the non-infected control group and weakening of potential associations. On the other hand, individuals with long COVID may exhibit greater symptom awareness, which could have led to an over-reporting of sleep problems and, consequently, to an inflated observed association. Therefore, the results should be interpreted with caution. Furthermore, analyses were conducted using a weighting factor; however, selection bias due to non-response cannot be completely ruled out. It is reasonable to assume that patients severely affected by long COVID or with ME/CFS did not participate in the study, which limits the representativeness of the data. Individuals with severe long COVID, as well as those experiencing severe sleep problems, may have difficulty participating in a telephone interview and providing detailed information. This could have led to a systematic bias towards the underestimation of the reported prevalence and effect sizes.

## 5 Conclusion

Our study provides insights into the association between SARS-CoV-2 infection, long COVID status, sleep problems and sleep quality among adults in Germany. Individuals with current long COVID reported more frequent sleep problems and poorer sleep quality than those without SARS-CoV-2 infection or without a history of long COVID. These associations persisted after adjusting for sociodemographic, health-related, and SARS-CoV-2 associated indicators. Taken together, the findings suggest that sleep problems constitute a relevant burden among adults with current long COVID, in particular with regard to problems maintaining sleep and poor sleep quality. Given the negative consequences for physical and mental health, our findings suggest that more attention should be paid to distinguishing between different types of sleep problems in clinical assessment and management of patients with long COVID. At the same time, the occurrence of sleep problems in individuals without SARS-CoV-2 infection highlights the need of interpreting sleep problems within a broader health and psychosocial context.

## Data Availability

The datasets presented in this article are not readily available because the informed consent of the study participants does not cover the public provision of the data. The raw data supporting the conclusions of this article is archived at the Robert Koch Institute’s Research Data Center and can be viewed by researchers on reasoned request. Data access is possible on site in the Secure Data Center of the Research Data Center of the Robert Koch Institute. Requests to access the datasets should be directed to: fdz@rki.d.
