# Supplementary material for: Self-reported long COVID associated with sleep problems and poor sleep quality among adults in Germany: results of the German Health Update 2023 (GEDA 2023)
Source: Front Public Health. 2026 Oct 14;14:1814427. doi: 10.3389/fpubh.2026.1814427 (PMC13713641; doi:10.3389/fpubh.2026.1814427)
Supplement: Supplementary file 1 [file Supplementary_file_1.docx]

**Supplementary Material**

Table S1. Weighted distribution of ordinal responses for sleep variables

|  | ***n*** | **% (95% CI)** |
| --- | --- | --- |
| **Sleep onset problems** | | |
| Not at all during the past 4 weeks | 4,245 | 44.0 (42.4–45.6) |
| Less than once a week | 1,968 | 19.8 (18.6–21.1) |
| Once or twice a week | 1,739 | 18.4 (17.1–19.7) |
| Three times a week or more | 1,358 | 17.8 (16.4–19.1) |
| **Problems maintaining sleep** | | |
| Not at all during the past 4 weeks | 3,350 | 39.8 (38.2–41.5) |
| Less than once a week | 1,501 | 14.4 (13.3–15.5) |
| Once or twice a week | 1,732 | 17.4 (16.2–18.6) |
| Three times a week or more | 2,727 | 28.4 (26.9–29.9) |
| **Subjective sleep quality** | | |
| Very good | 1,572 | 16.7 (15.4–17.9) |
| Quite good | 5,804 | 59.8 (58.1–61.4) |
| Quite poor | 1,626 | 19.2 (17.9–20.6) |
| Very poor | 308 | 4.3 (3.6–5.1) |

Table S2. Sample characteristics stratified by SARS-CoV-2 infection and long COVID status

|  | **No infection**  (*n*=3,470) | | **Infection without long COVID** (*n*=4,732) | | **Previous long COVID** (*n*=566) | | **Current long COVID** (*n*=542) | |
| --- | --- | --- | --- | --- | --- | --- | --- | --- |
|  | ***n*** | **% (95% CI)** | ***n*** | **% (95% CI)** | ***n*** | **% (95% CI)** | ***n*** | **% (95% CI)** |
| **Age groups** | | | | | | | | |
| 18–29 years | 181 | 11.1 (9.1–13.2) | 511 | 20.6 (18.4–22.7) | 67 | 18.2 (12.6–23.8) | 33 | 11.2 (5.8–16.6) |
| 30–44 years | 260 | 14.7 (12.3–17.0) | 911 | 26.7 (24.5–28.8) | 123 | 30.0 (23.5–36.5) | 80 | 25.7 (18.9–32.5) |
| 45–64 years | 988 | 30.9 (28.4–33.4) | 1,961 | 36.5 (34.4–38.6) | 196 | 29.3 (23.6–35.0) | 268 | 44.9 (37.9–51.8) |
| 65–79 years | 1,255 | 26.8 (24.7–29.0) | 1,051 | 12.8 (11.6–14.0) | 139 | 17.9 (13.2–22.6) | 112 | 11.3 (8.2–14.3) |
| 80 years and older | 786 | 16.5 (14.7–18.2) | 298 | 3.5 (2.8–4.1) | 41 | 4.6 (2.6–6.7) | 49 | 6.9 (3.9–10.0) |
| **Sex** | | | | | | | | |
| Female | 1,832 | 47.1 (44.4–49.8) | 2,451 | 51.8 (49.5–54.1) | 331 | 54.8 (48.1–61.5) | 330 | 60.5 (53.5–67.4) |
| Male | 1,638 | 52.9 (50.2–55.6) | 2,281 | 48.2 (45.9–50.5) | 235 | 45.2 (38.5–51.9) | 212 | 39.5 (32.6–46.5) |
| **Level of education (CASMIN)** | | | | | | | | |
| Low | 741 | 34.7 (32.0–37.5) | 506 | 20.3 (18.2–22.5) | 87 | 25.0 (18.9–31.1) | 95 | 31.9 (24.9–38.9) |
| Medium | 1,519 | 51.0 (48.2–53.7) | 2,009 | 55.4 (53.1–57.7) | 241 | 54.9 (48.4–61.5) | 266 | 54.1 (47.1–61.1) |
| High | 1,210 | 14.3 (13.0–15.6) | 2,217 | 24.3 (22.7–25.8) | 238 | 20.1 (15.8–24.3) | 181 | 13.9 (10.9–17.0) |
| **Chronic condition** | | | | | | | | |
| Yes | 2,009 | 56.5 (53.8–59.3) | 2,200 | 43.1 (40.8–45.3) | 309 | 54.6 (48.0–61.2) | 415 | 76.3 (70.1–82.4) |
| No | 1,461 | 43.5 (40.7–46.2) | 2,532 | 56.9 (54.7–59.2) | 257 | 45.4 (38.8–52.0) | 127 | 23.7 (17.6–29.9) |
| **Depressive symptoms** | | | | | | | | |
| Yes | 574 | 20.9 (18.6–23.3) | 454 | 11.8 (10.2–13.4) | 75 | 18.8 (13.4–24.2) | 157 | 33.8 (27.2–40.4) |
| No | 2,896 | 79.1 (76.7–81.4) | 4,278 | 88.2 (86.6–89.8) | 491 | 81.2 (75.8–86.6) | 385 | 66.2 (59.6–72.8) |
| **COVID-19 vaccination status** | | | | | | | | |
| Yes | 3,195 | 88.8 (86.8–90.9) | 4,440 | 91.3 (89.9–92.7) | 533 | 92.0 (88.2–95.8) | 525 | 96.8 (94.4–99.2) |
| No | 275 | 11.2 (9.1–13.2) | 292 | 8.7 (7.3–10.1) | 33 | 8.0 (4.2–11.8) | 17 | 3.2 (0.8–5.6) |
| **Multiple SARS-CoV-2 infections** | | | | | | | | |
| Yes | 0 | 0.0 (0.0–0.0) | 1,066 | 26.0 (23.9–28.1) | 190 | 34.4 (28.2–40.5) | 186 | 32.5 (26.0–39.0) |
| No | 3,470 | 100.0 (100.0–100.0) | 3,666 | 74.0 (71.9–76.1) | 376 | 65.6 (59.5–71.8) | 356 | 67.5 (61.0–74.0) |

*Note*. CASMIN=Comparative Analysis of Social Mobility in Industrial Nations.

Table S3. Frequency and weighted percentage of participants with all three sleep problems, according to SARS-CoV-2 infection and long COVID status

| **Group** | ***n*** | **% (95% CI)** |
| --- | --- | --- |
| No infection | 354 | 11.8 (10.1–13.7) |
| Infection without long COVID | 286 | 7.2 (6.0–8.5) |
| Previous long COVID | 54 | 10.0 (6.8–14.6) |
| Current long COVID | 105 | 22.6 (16.7–29.7) |

Table S4. Effect estimates from logistic regression analyses including all covariates

| **Variable** | **Sleep onset problems** | | **Problems maintaining sleep** | | | **Poor sleep quality** | |  |
| --- | --- | --- | --- | --- | --- | --- | --- | --- |
|  | **aOR (95% CI)** | ***p*** | **aOR (95% CI)** | | ***p*** | **aOR (95% CI)** | ***p*** |  |
| **SARS-CoV-2 infection and long COVID status** | | | | | | | | |
| No infection | Ref. |  | | Ref. |  | Ref. |  |  |
| Infection without long COVID | 0.79 (0.62–0.99) | 0.043 | | 0.92 (0.77–1.09) | 0.333 | 0.87 (0.71–1.07) | 0.182 |  |
| Previous long COVID | 1.09 (0.74–1.60) | 0.670 | | 1.11 (0.77–1.58) | 0.575 | 1.06 (0.73–1.53) | 0.751 |  |
| Current long COVID | 1.38 (0.92–2.05) | 0.118 | | 1.73 (1.23–2.44) | 0.002 | 1.81 (1.29–2.55) | <0.001 |  |
| **Sex** | | | | | | | | |
| Male | Ref. |  | | Ref. |  | Ref. |  |  |
| Female | 1.54 (1.26–1.88) | <0.001 | | 1.36 (1.16–1.58) | <0.001 | 1.36 (1.14–1.62) | <0.001 |  |
| **Age groups** | | | | | | | | |
| 18–29 years | Ref |  | |  |  |  |  |  |
| 30–44 years | 0.82 (0.57–1.18) | 0.281 | | 2.11 (1.48–3.01) | <0.001 | 1.46 (1.04–2.03) | 0.028 |  |
| 45–64 years | 0.81 (0.59–1.11) | 0.191 | | 2.79 (2.04–3.84) | <0.001 | 1.38 (1.03–1.86) | 0.033 |  |
| 65–79 years | 0.61 (0.43–0.87) | 0.006 | | 2.53 (1.81–3.53) | <0.001 | 0.83 (0.60–1.16) | 0.275 |  |
| 80 years and older | 0.71 (0.48–1.05) | 0.088 | | 3.13 (2.16–4.56) | <0.001 | 0.70 (0.46–1.05) | 0.084 |  |
| **Level of education (CASMIN)** | | | | | | | | |
| Low | Ref. |  | | Ref. |  | Ref. |  |  |
| Medium | 0.67 (0.52–0.85) | 0.001 | | 0.85 (0.69–1.04) | 0.107 | 0.91 (0.73–1.14) | 0.405 |  |
| High | 0.40 (0.31–0.53) | <0.001 | | 0.77 (0.62–0.95) | 0.013 | 0.76 (0.60–0.96) | 0.021 |  |
| **Chronic condition** | | | | | | | | |
| No | ref. |  | | Ref. |  | Ref. |  |  |
| Yes | 2.13 (1.71–2.66) | <0.001 | | 1.60 (1.36–1.88) | <0.001 | 1.72 (1.43–2.07) | <0.001 |  |
| **Depressive symptoms** | | | | | | | | |
| No | Ref. |  | | Ref. |  | Ref. |  |  |
| Yes | 3.28 (2.60–4.14) | <0.001 | | 2.60 (2.11–3.21) | <0.001 | 3.57 (2.91–4.39) | <0.001 |  |
| **COVID-19 vaccination status** | | | | | | | | |
| Yes | Ref. |  | | Ref. |  | Ref. |  |  |
| No | 0.90 (0.63–1.28) | 0.550 | | 1.08 (0.80–1.45) | 0.636 | 1.01 (0.75–1.36) | 0.959 |  |
| **Multiple SARS-CoV-2 infections** | | | | | | | | |
| No | Ref. |  | | Ref. |  | Ref. |  |  |
| Yes | 1.01 (0.76–1.33) | 0.972 | | 1.06 (0.84–1.34) | 0.639 | 0.95 (0.74–1.22) | 0.712 |  |

*Note*. CASMIN=Comparative Analysis of Social Mobility in Industrial Nations.

**Sensitivity analysis**

Table S5. Weighted prevalence estimates of sleep onset problems, problems maintaining sleep and poor sleep quality overall and varying by SARS-CoV-2 infection and self-reported medically diagnosed long COVID status, as well as effect estimates from logistic regression analyses (N = 8,929)

|  | **Prevalence** | | **Effect estimates** | | | |
| --- | --- | --- | --- | --- | --- | --- |
|  | ***n*** | ***%* (95% CI)** | **OR (95% CI)** | ***p*** | **aOR (95% CI)** | ***p*** |
| **Sleep onset problems** | **1,311** | **17.9 (16.5–19.3)** |  | |  | |
| No infection | 587 | 20.0 (17.8–22.3) | Ref. |  | Ref. |  |
| Infection without long COVID | 655 | 15.6 (14.0–17.4) | 0.74 (0.61–0.90) | 0.003 | 0.86 (0.68–1.07) | 0.175 |
| Previous long COVID | 29 | 24.3 (14.1–38.6) | 1.29 (0.65–2.54) | 0.468 | 1.21 (0.61–2.41) | 0.593 |
| Current long COVID | 40 | 46.2 (30.0–63.3) | 3.45 (1.71–6.94) | <0.001 | 2.24 (0.93–5.35) | 0.071 |
| **Problems maintaining sleep** | **2,620** | **28.3 (26.8–29.8)** |  | |  | |
| No infection | 1,094 | 30.8 (28.4–33.4) | Ref. |  | Ref. |  |
| Infection without long COVID | 1,417 | 26.0 (24.1–27.9) | 0.79 (0.67–0.92) | 0.002 | 1.00 (0.84–1.19) | 0.995 |
| Previous long COVID | 51 | 29.9 (20.0–42.1) | 0.96 (0.56–1.65) | 0.873 | 0.92 (0.50–1.71) | 0.801 |
| Current long COVID | 58 | 57.0 (39.6–72.8) | 2.97 (1.47–5.99) | 0.002 | 2.26 (1.11–4.61) | 0.025 |
| **Poor sleep quality** | **1,852** | **23.5 (22.1–25.1)** |  | |  | |
| No infection | 729 | 24.1 (21.8–26.6) | Ref. |  | Ref. |  |
| Infection without long COVID | 1,040 | 22.5 (20.6–24.4) | 0.91 (0.77–1.08) | 0.285 | 0.96 (0.79–1.17) | 0.719 |
| Previous long COVID | 34 | 24.2 (15.1–36.5) | 1.00 (0.55–1.82) | 0.987 | 0.88 (0.46–1.69) | 0.709 |
| Current long COVID | 49 | 53.4 (36.6–69.5) | 3.61 (1.81–7.18) | <0.001 | 2.25 (1.07–4.76) | 0.033 |

*Note.* OR=odds ratio; aOR=adjusted odds ratio, adjusted for age group, sex, level of education, chronic conditions depressive symptoms, COVID-19 vaccination status and multiple SARS-CoV-2 infections. In the sensitivity analysis, long COVID status is based on medically diagnosed long COVID only. Participants who reported subjective long COVID without a medical diagnosis were therefore reclassified into "Infection without long COVID" rather than excluded (n = 733; 356 from the previous- and 377 from the current-long-COVID groups of the main analysis). Group sizes: No infection: n = 3,470; Infection without long COVID: n = 5,184; previous long COVID: n = 168; current long COVID: n = 107.
